# The Effect of Place of Residence on Treatment Outcomes and Survival in Octogenarian and Nonagenarian Breast Cancer Patients

**DOI:** 10.7759/cureus.11934

**Published:** 2020-12-06

**Authors:** Hasan Dagmura, Emin Daldal

**Affiliations:** 1 Surgical Oncology, Kütahya Health Sciences University Evliya Çelebi Training and Research Hospital, Kütahya, TUR; 2 General Surgery, Gaziosmanpasa University, Tokat, TUR

**Keywords:** age, breast, elderly, octogenarian, surgery

## Abstract

Introduction

The number of octogenarian invasive breast cancer cases is projected to increase, as there is a significant increase in life expectancy. However, no specific treatment guideline has been established so far for this vulnerable group of patients. The aim of the present study was to evaluate the treatment outcomes of octogenarians diagnosed with early and locally advanced invasive breast cancer, to compare those who underwent surgery with conventional treatment and those who did not, and to reveal the potential social factors that may affect their therapy outcomes.

Material and methods

A total of 78 patients aged 80 and over were included in the study. There was a significant relationship between a patient’s social milieu and treatment status (p < 0.001). The relationship between receiving endocrine therapy or surgical treatment was also significant (p = 0.029).

Results

The surgical treatment rate was 90.9% in survivors, which was significantly lower in those who passed away (37.8%, p < 0.001). According to the log-rank test results, life expectancy was significantly longer in operated patients than in non-operated ones (p < 0.001). The median survival length was 62 months (range: 33.8-90.2) in operated patients 80 years of age and above and 19 months (range: 16.3-21.7) in non-operated ones. The surgical treatment frequency was 15.30 times (range: 4.86-48.21) higher in patients living with family than in patients living alone or in a nursing home.

Conclusion

Thus, the social milieu of the patients, especially the place of residence, had a major impact on the treatment of the elderly (octogenarians) patients with breast cancer. Surgery and endocrine therapy as an adjuvant treatment were tolerable and had positive impacts on survival.

## Introduction

The considerable increase in life expectancy has led to an enlargement of the elderly population. It is known that cancer incidence rises with age and that the most common malignancy in women is breast cancer. Thus, a considerable increase is expected in the number of elderly patients with breast cancer. Indeed, the National Cancer Institute (NCI) has declared that the number of invasive breast cancer cases is projected to double by 2030. Since there are no breast cancer screening programs for women over the age of 70 and only individuals with good health status are screened by mammography, elderly patients usually tend to present with more advanced stages of the disease as compared to younger ones, precluding them from the unnecessary stereotactic biopsy [1]. Moreover, at advanced stages, they are more vulnerable to be undertreated since they need more invasive treatment [2].

Despite this alarming increase in the number of elderly breast cancer patients, no well-defined protocols or treatment strategies have been adopted for this vulnerable group so far. Furthermore, this group of patients receives a wider variety of treatment programs as compared to younger patients. This fact is mainly due to age-related biases [3]. It is also worth noting that this group of the elderly population is underrepresented in most studies conducted [4-5], and this is explained by mentioning the erroneous belief that this patient group represents a minority of the population. The possible reasons for undertreating these elderly patients are that the tumor biology in the elderly is relatively less aggressive than in younger patients, the elderly patients tend not to tolerate chemotherapy due to its related toxicities, and that the elderly people do have other comorbidities with a higher risk of death than breast cancer itself [6].

The aim of the present study was to evaluate the treatment outcomes in octogenarian patients with early- and locally advanced stage invasive breast cancer who underwent surgery with conventional treatment and those who did not and to reveal potential social factors that may explain under-treatment or even refusal of treatment in this group of patients.

## Materials and methods

**Figure 1:**
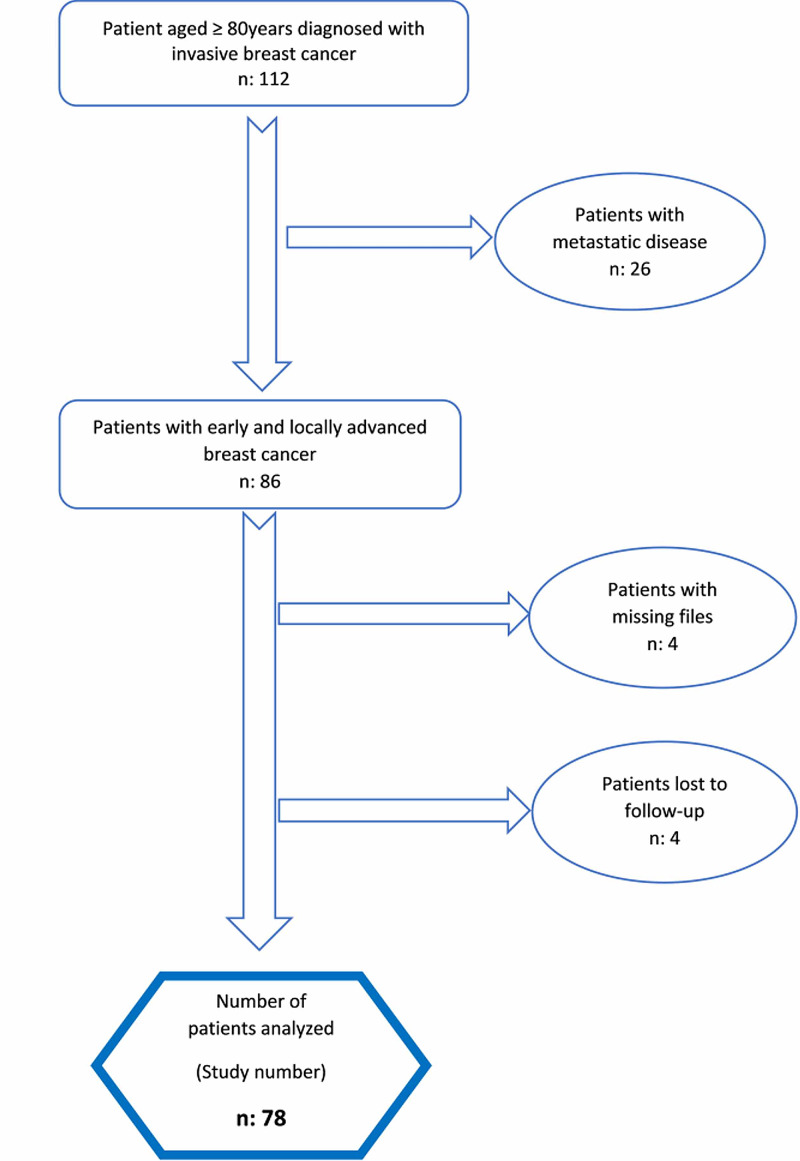
Flowchart of study patients

This is a prospective observational cohort study designed for the evaluation of consecutive cases collected in a single tertiary academic referral center. Ethical committee approval was obtained for the study from the Local Research Ethics Committee of Tokat Gaziosmanpaşa University, School of Medicine (19-KAEK-022). The patients were recruited after reviewing the electronic database using the relevant International Classification of Diseases 10th Revision (ICD-10) diagnosis codes. The search was limited to the period between January 2009 and June 2017. Demographic and clinical data of octogenarians and nonagenarians (i.e. patients ≥ 80 years of age), comorbidities, treatments offered (surgery, radiotherapy, chemotherapy, and other therapies), treatments received, the major factor affecting the treatment choice, Eastern Cooperative Oncology Group (ECOG) scores, the place of residence (alone, with family members, spouse or partner, or in a nursing home), and pathologic and operative reports were retrieved from the electronic medical files and recorded, including the receptor status (hormone receptor-positive breast cancer patients are considered to have either estrogen (ER) or progesterone (PR) receptors or both). Patients with missing or incomplete medical files and those with whom contact was lost for follow-ups were excluded from the study. Patients with metastatic disease, patients at stage “0,” and patients with ductal carcinoma in situ were also excluded (Figure 1).

The primary outcomes of interest were to analyze factors that may affect the decision-making process in the treatment of octogenarians with breast cancer. The secondary outcome of interest was to compare survival differences between octogenarians treated with surgery and adjuvant therapy and their counterpart who were undertreated or who did not receive any treatment.

Statistical methods

Statistical analyses were performed using SPSS software (Version 22.0, IBM Corp., Armonk, NY). Descriptive statistics of continuous variables were presented as mean ± standard deviation or median (min-max) depending on the normality of the distribution. Descriptive statistics of categorical variables were given as numbers and percentages (%). The normality of distribution was examined by the Shapiro-Wilk test. Means of the two independent groups were compared by the student's t-test. Categorical variables were evaluated with chi-square or Fisher's exact tests. The Kaplan-Meier test was used to assess the relationship between treatment status and patients' overall survival, and the log-rank test was used to compare Kaplan-Meier survival curves for each group. Univariate and multivariate binary logistic regression analyses were used to determine the factors affecting the treatment status. According to the univariate analysis, all variables associated with the treatment status at a significance level of < 0.05 were included in the multivariate model. Odds ratio (OR) and 95% confidence interval (CI) values were calculated for each significant parameter in the univariate and final multivariate models. p < 0.05 was considered the lowest level of statistical significance.

## Results

A total of 78 patients aged 80 and over were included in the study. The mean age of the patients was 82.83 ± 2.88 years (range: 80-93). Out of 78 patients, 47 (60.3%) were operated on while 31 (39.7%) did not have surgery. There was no significant difference between the mean ages of the operated (82.62 ± 2.91 years) and non-operated patients (83.16 ± 2.85 years) (p = 0.418). By the end of the study (last day of the study), 45 of the patients (57.7%) had passed away and 33 (42.3%) were alive. The mean tumor diameter of the patients was 3.50 ± 1.27 cm (range: 1.2-9.0). The mean tumor diameter of the treated (3.46 ± 1.52 cm) and non-treated patients (3.56 ± 0.77 cm) were similar (p = 0.696). Other socio-demographic and clinical characteristics of the patients are presented in Table 1.

**Table 1:** Descriptive statistics for socio-demographic and clinical characteristics of patients 80 years old and above (n=78) ECOG: Eastern Cooperative Oncology Group, MRM: modified radical mastectomy, BCS: breast-conserving surgery

Variables	Groups	Frequency (%)
Surgery type	None	31 (39.7)
	MRM	37 (47.4)
	BCS+ axillary dissection	9 (11.5)
	Simple mastectomy	1 (1.3)
Breast side	Right	44 (56.4)
	Left	34 (43.6)
Status at the end of the study	Alive	33 (42.3)
	Exitus	45 (57.7)
Place of residence	Alone	6 (7.7)
	Family	46 (59)
	Nursing home	26 (33.3)
ECOG score	0	7 (9)
	1	34 (43.6)
	2	34 (43.6)
	3+4	3 (3.8)
Stage	1b	2 (2.6)
	2a	7 (9)
	2b	57 (73.1)
	3a	6 (7.7)
	3b	6 (7.7)
Receptor status	Positive	50 (64.1)
	Negative	28 (35.9)
Endocrine therapy	-	36 (46.2)
	+	42 (53.8)
Adjuvant chemo-radiotherapy	-	62 (79.5)
	+	16 (20.5)
Total		78 (100)

The relationships between the socio-demographic and other clinical characteristics of the patients and their treatment characteristics are presented in Table 2.

**Table 2:** Relationship between socio-demographic and clinical characteristics of the patients and their surgical treatment status * Statistically significant (p<0.05) ^a^Chi square test ^b^Fisher exact test

	Groups	Surgical treatment n (%)			P-values
		No	Yes	Total	
Place of residence	Alone	6 (100)	0 (0)	6	<0.001^b^*
	Family	7 (15.2)	39 (84.8)	46	
	Nursing home	18 (69.2)	8 (30.8)	26	
Stage	1b	0 (0)	2 (100)	2	0.845^b^
	2a	2 (28.6)	5 (71.4)	7	
	2b	24 (42.1)	33 (57.9)	57	
	3a	2 (33.3)	4 (66.7)	6	
	3b	3 (50)	3 (50)	6	
ECOG score	0+1+2	30 (40)	45 (60)	75	1.000^b^
	3+4	1 (33.3)	2 (66.7)	3	
Endocrine therapy	No	19 (52.8)	17 (47.2)	36	0.029^a^*
	Yes	12 (28.6)	30 (71.4)	42	
Adjuvant chemo-radiotherapy	No	31 (50)	31 (50)	62	<0.001^a^*
	Yes	0 (0)	16 (100)	16	
Receptor status	Positive	16 (32)	34 (68)	50	0.062^a^
	Negative	15 (53.6)	13 (46.4)	28	
Breast side	Right	22 (50)	22 (50)	44	0.035^a^*
	Left	9 (26.5)	25 (73.5)	34	
Status	Alive	3 (9.1)	30 (90.9)	33	<0.001^a^*
	Exitus	28 (62.2)	17 (37.8)	45	

There was a significant relationship between the social milieu of the patients and their treatment status (p < 0.001). None of the patients who lived alone received surgical treatment. There was no significant relationship between the Eastern Cooperative Oncology Group (ECOG) scores and treatment status (p = 1.000). Sixty percent of the patients with ECOG level 0 + 1 + 2 were surgically treated. Similarly, 66.7% of the patients with ECOG level 3 + 4 had surgery. There was a significant relationship between receiving endocrine therapy and having surgical treatment (p = 0.029). While 71.4% of those who received hormone therapy had undergone surgery, only 47.2% of the patients who did not receive hormonal therapy had undergone surgery. Besides, there was a significant correlation between receiving surgical treatment and adjuvant chemo-radiotherapy (p < 0.001). All patients who received adjuvant chemo-radiotherapy treatment had been operated on, but only 50% of those who did not receive adjuvant chemo-radiotherapy had undergone surgical treatment.

There was a significant relationship between diseased breast (left or right) and surgical treatment frequency (p = 0.035). Surgical treatment was applied in 50% of the patients with tumors in the right breast while 73.5% of the patients with tumors in the left breast were operated on. The surgical treatment rate was 90.9% among the survivors while only 37.8% of the patients who passed away had been operated on (p < 0.001). Among a total of 42 patients who had endocrine treatment (positive receptors), 30 patients had also received surgical treatment. Of them, 86.7% were living with their families while the rest of them were living in nursing homes. Three of the 12 patients (25%) who did not have surgical treatment were living with their families. In addition, 13.3% of the 30 patients who had undergone surgical treatment were living in a nursing home while 66.7% of the 12 patients who had not received surgical treatment were living in a nursing home. Of the 12 patients who had not received surgical treatment before the endocrine therapy, 25% were living with their families, 66.7% in nursing homes, and 8% alone (Table 3).

**Table 3:** The relationship between surgical treatment status and living conditions of patients with receptor-positive who had received endocrine therapy * Statistically significant (p<0.001) ^b^Fisher exact test

			Place of residence			Total	P value	
			Alone	Family	Nursing home			
Endocrine therapy (yes)	Surgical treatment	No	1 (8.3)	3 (25.0)	8 (66.7)	12 (100)	<0.001^a^*	
								Yes	0 (0)	26 (86.7)	4 (13.3)	30 (100)	
		Total		1 (2.4)	29 (69.0)	12 (28.6)							42 (100)		

**Figure 2:**
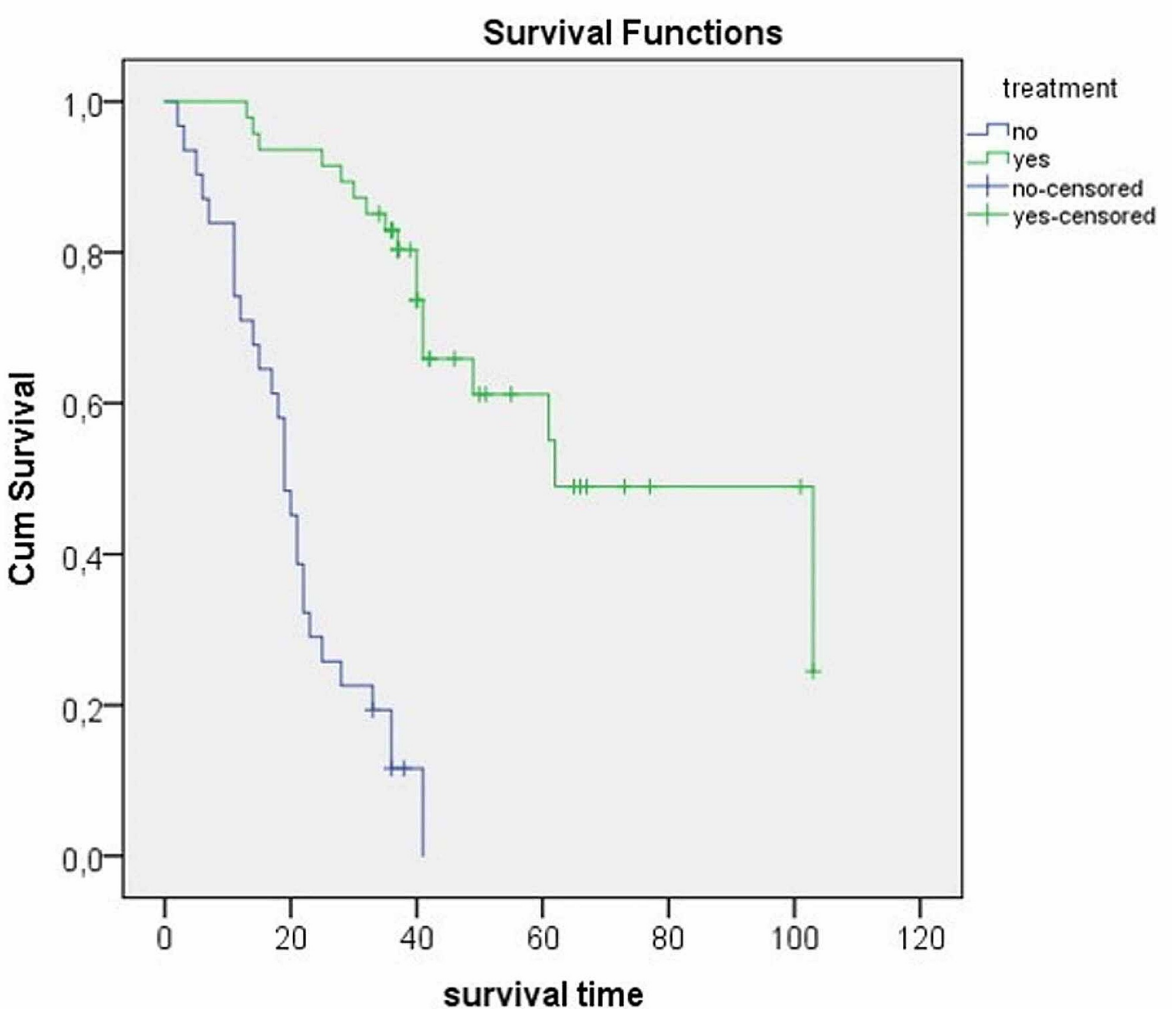
Comparison of survival curves of patients aged 80 years and above by treatment status

The life expectancies and survival plots of the patients who had and did not have surgical treatment were calculated by the Kaplan-Meier test and are presented in Table 4. According to the log-rank test results, the life expectancy of the operated patients was significantly higher than that of non-operated ones (p < 0.001; Figure 2).

**Table 4:** Means and medians for survival time according to surgical treatment status * Kaplan-Meier survival analysis with the log-rank (Mantel-Cox) test

	Median			Mean			
	Estimate (month)	95% Confidence Interval		Estimate (month)	95% Confidence Interval		P values
		Lower Bound	Upper Bound		Lower Bound	Upper Bound	
Surgical treatment							
No	19	16.3	21.7	20.6	16.5	24.7	<0.001*
Yes	62	33.8	90.2	71.2	58.8	83.5	

The median length of survival was 62 months in patients aged 80 years and over (range: 33.8-90.2), whereas non-operated patients had a median survival length of 19 months (range: 16.3-21.7) (Table 4).

According to the results of univariate test analysis performed to determine the factors affecting patients’ likelihood of receiving surgical treatment, the place of residence, and endocrine treatment variables turned out to be statistically significant (p < 0.05). The variables that turned out to be significant in the univariate model were included in the multivariate model. Non-significant variables of ECOG score, receptor status, adjuvant chemo-radiotherapy, clinical/pathologic stage, and tumor diameter were not included in the multivariable model. According to the multivariate model, only the patients’ place of residence variable had a significant influence on the likelihood of receiving surgical treatment (p < 0.001). On the other hand, the likelihood of receiving endocrine therapy was not significantly associated with undergoing surgery (p = 0.204). The probability of a patient living with a family to receive surgical treatment was 15.30 times higher (range: 4.86-48.21) than a patient living alone or living in a nursing home (p < 0.001, Table 5).

**Table 5:** Univariate and multivariate binary logistic regression analyses for surgical treatment status Reference category for ECOG (Eastern Cooperative Oncology Group): 3+4 Reference category for living place: Alone + Nursing home Reference category for receptor status: Negative Reference category for adjuvant chemo-radiotherapy: No Reference category for hormonal treatment: No Reference category for stage: 1b+2a ns: not significant, CI: confidence interval

	Univariate		Multivariate	
	P values	Odds ratio (CI 95%)	P values	Odds ratio (CI 95%)
Place of residence				
(Family)	<0.001*	16.71 (5.37-51.98)	<0.001*	15.30 (4.86-48.21)
Endocrine therapy				
(Yes)	0.031*	2.794 (1.09-7.12)	ns (0.204)	-
ECOG score				
(0+1+2)	0.818		not included	-
Receptor status				
(Positive)	0.065		not included	-
Adjuvant chemo-radiotherapy				
(Yes)	0.998		not included	-
Stage				-
2b	0.269		not included	
3a	0.635			
3b	0.274			
Tumor diameter (cm)	0.726		not included	-
Multivariable: Nagelkerke R Square = 0.446, Classification: 80.8%				

## Discussion

A spectacular advancement has been made in the field of molecular oncology by moving from standard treatment to the introduction of the new personalized treatment concept, which optimizes the patient-specific treatment according to the molecular composition of individual tumors. Different biological subtypes of breast cancer were identified through the use of gene expression profiling (viz. Luminal A, Luminal B, Her/neu (+) enriched and basal-like subgroups) and a different therapy program has been developed for each group [7-8]. However, despite this development, psychological, physical, and social aspects of patients’ stories are either underestimated or not even taken into consideration. ECOG and Karnofsky scales were developed a few decades ago to determine the suitability of patients for chemotherapy, radiotherapy, surgery, or other treatment modalities [9-10]. Where ECOG score consists of a simple performance-status scale to quantify the patient's general well-being and activities of daily life, runs from 0 to 5, with 0 denoting perfect health and 5 death. However, these scales are vulnerable to subjectivity and have high interobserver variability [11-12]. Hence, they have not been sufficient so far to evaluate the suitability of patients to undergo a systemic treatment. Indeed, in the present study, no correlation was found between the ECOG score and the treatment status of the patient, i.e., whether the patient received surgical and complementary treatments.

Interestingly, a significant relationship was found between patients’ place of residence and the received guideline-specified therapy. None of the six patients living alone received surgical treatment even though they were scheduled for surgery since they were in the early stages of breast cancer and had ECOG scores between 0 and 2. Furthermore, out of 42 patients who had endocrine therapy (ET), 30 patients had previously received surgery. Twenty-six of these 30 patients (86.7%) were living with their families. Of the 12 patients who had not undergone surgery but only received ET, three (25%) were living alone, eight (66.7%) were living in a nursing home, and one (8%) was living alone. This finding brings to the fore the fact that the social milieu of a patient has an important impact on the probability of receiving treatment. The support from a patient’s spouse, children, or other family members is a crucial element affecting the decision-making during the treatment process. This social factor had a major influence on the treatment and was an independent prognostic factor for the likelihood of receiving guideline-specified therapy in these physically and psychosocially fragile populations.

The fundamental treatment of early-stage breast cancer is surgery. Surgical options are: 1) modified radical mastectomy (MRM), 2) breast-conserving surgery (BCS) and sentinel lymph node (SLB) when axillary staging is indicated, or 3) simple mastectomy. BCS + SLB became popular since it is advantageous over MRM in terms of morbidities such as edema in the arm, axillary region numbness, decreased range of motion, and pain [13-16]. The operative mortality rate in the present study was 2%, which was similar to the mortality rates reported by many other studies [17]. Breast surgery is a well-tolerated procedure even in elderly patients. A study conducted by Petkke et al. showed that breast surgery is still safe for patients over 80 years of age with low postoperative morbidity and mortality rates [18]. In patients with severe comorbidities where general anesthesia is risky, lumpectomy or even MRM is still feasible under local anesthesia and regional nerve blocks [19]. In the present study, 78.7% of the elderly preferred MRM to BCS (18.3%), and this preference could be attributed to their desire to avoid radiotherapy. An alternative treatment to radiotherapy in patients with early breast cancer who were receptor-positive and underwent BCS is ET with tamoxifen or AIs [20].

Patients with early-stage receptor-positive breast cancer are often scheduled to receive ET as an adjuvant treatment. This is because tamoxifen has been proven to reduce annual breast cancer death and recurrence rates irrespective of axillary nodal status. Compared to tamoxifen, AIs extend the length of disease-free survival period and have lower recurrence rates especially in the first year of treatment [21]. Receptor-positive patients with severe comorbidities or those who refuse surgery can be given ET as a primary treatment. Chemotherapy, another treatment option as adjuvant therapy, has been proven to cause severe toxicity in the elderly [22]. Therefore, it is specially reserved for receptor-negative, axillary node-positive, fit patients. Human epidermal growth factor receptor (HER)-directed therapy has recently been introduced as a treatment option in combination with chemotherapy for HER-positive patients.

The results of a study conducted by Owusu et al. showed that up to 66% of women older than 75 years received less than guideline-specific therapy [23]. Moreover, a study conducted by Van der Water et al. showed that 55.6% of early breast cancer patients were not treated in accordance with guidelines [24]. A similar ratio (55.1%) was found in the present study. Besides this high rate of discordance in therapy, a significant correlation was found between receiving ET and surgical treatment. Indeed, 71.4% of those who had ET had already undergone surgical treatment in the form of MRM, breast-conserving surgery+/- SLND, or simple mastectomy. Similarly, in the present study, a statistically significant relationship was found between receiving adjuvant chemo-radiotherapy and receiving surgical treatment. In fact, all patients who received adjuvant therapy had undergone surgery (p-value < 0.001). This could be explained by the vital importance of the decision-making process to persuade patients to undertake the planned treatment. This could be achieved by the combination of multidisciplinary care, family support, psychosocial support, and communication with patients. Once they started, the patients living with their families tended to accomplish the treatment protocol. Similarly, treatment consistency was poor, especially in the patient group who had not received surgery, suggesting that those who had not undergone surgery were also more inclined to refuse drug therapy.

In this cohort, not only tumor-related factors or the patient’s physical status but also the psychosocial status of the patients significantly influenced their overall survival rate. Indeed, patients in their eighties and nineties with early and locally advanced-stage breast cancer living with their families had a much higher chance of completing conventional treatment for breast cancer (84.8%). On the other hand, those with the same stage of the disease, similar tumor characteristics, and similar ECOG score (0-2) but living in a nursing home or alone were unlikely to receive the conventional treatment since only 25% of them had surgery +/- adjuvant treatment. In other words, the place of residence had a significant influence on the likelihood of receiving surgical treatment. The probability of a patient living with a family to receive surgical treatment was 15.3 times higher than that of a patient living alone or living in a nursing home. This could be explained by the social bonds existing among family members and their strong commitment to supporting their beloved sick relative. The family members feel obliged to take care of their sick relatives and consider this a duty toward the sick relative just as the sick one did when he/she was in good health. On the other hand, the nursing home workers feel that he/she has to simply fulfill his/her planned task without the obligation to persuade these patients to stick to their treatment plan or to show special emotional attention to them.

According to the results of the present study, the ECOG score alone does not reflect a patient’s health status. Two patients with the same ECOG score, one staying with the family and receiving full emotional and psychosocial support cannot have the same mental and physical health status as a patient living alone or in a nursing home since the morale of a patient plays an important hidden role in the treatment outcome. Therefore, while evaluating the general status of a patient, the social factor, especially the place of residence, should also be incorporated into the evaluation system along with general status evaluating systems such as ECOG and Karnofsky scores.

Two factors were found to be independent predictors of the likelihood of a patient to receive guideline-specific therapy, i.e. surgery, the place of residence, and hormonal receptor-positivity. Of the patients who were alive on the last day of the study, 90.9% had undergone surgical treatment, whereas the surgical treatment rate was only 37.8% among those who passed away. Furthermore, the life expectancy of surgically treated patients was significantly higher than that of non-operated patients (62 vs. 19 months). This fact stressed the importance of surgical treatment and its superiority to other treatments in terms of survival in octogenarians and older patients. In fact, a trial conducted by Fentiman et al. showed that surgery (MRM) had a significant difference in progression-free survival as compared to primary endocrine therapy with tamoxifen alone [25].

The elderly who require long-term supportive care usually receive it from family members. However, when families are unable to provide this care, the most probable place of residence is a nursing home (NH). Besides the basic needs, nursing homes provide daily medical and social assistance. The elderly patients living alone or in NH are often unable to participate effectively in making decisions for their own medical care, especially in case of a devastating illness such as cancer, which may compromise their health status rapidly. In such a case, a care provider in the NH is needed to make the decision on behalf of them especially when the patient has a cognitive impairment.

The one-year mortality rate due to breast cancer surgery among nursing home residents was reported to be up to 31% [26], which was 12.5% in the surgical cohort of the present study. In addition, elderly patients are at high risk of functional deterioration not only during hospitalization but even after hospital discharge [27]. These patients still do need help and attention after surgery to overcome common unpleasant postoperative experiences such as pain, movement restriction, and depression. Patients living in an NH are at higher risk of mortality and functional decline during the first postoperative year [26].

In contrast to many studies reporting that breast cancer is more frequent in the left breast than in the right [28], the right breast was more often affected in the present study (56.4 vs 43.6%). In 73.5% of the cases, tumors in the left breast were treated while the right breast was treated in 50% of the cases. Assuming that the majority of the patients were right-handed, this finding could be explained by the refusal of the patient to undergo surgery due to the fear and worry that right arm pain and motility may be restricted after surgery, which could limit their daily activities. In fact, as much as one-third of non-elderly women treated surgically for breast cancer reported a diminished range of motion or pain in the sixth postoperative month [29-30].

The limitations of the present study include its methodology of an observational cohort study and the single institution factor that may cause unintended influence. Besides, patients with early and locally advanced breast cancer treated at our tertiary center might not represent the average surgical treatment outcomes in other healthcare institutions. An additional possible limitation might be due to a selection bias involving the fact that we studied only those patients who were scheduled for surgical treatment who were either at an early stage or with locally advanced disease. Another possible limitation of this study is that we studied breast cancer in the female population only.

## Conclusions

The social factor, precisely the place of residence, had a major effect in the treatment of elderly (octogenarians) patients with breast cancer. Surgery and ET as an adjuvant treatment were tolerable and have a positive impact on survival in this frail patient group.

## References

[1] Hacim NA, Akbaş A (2020). Stereotactic biopsy results of a series of patients with nonpalpable breast lesions in our hospital. Anatol Clin.

[2] Mustacchi G, Cazzaniga M, Pronzato P, De Matteis A, Di Costanzo F, Floriani I (2007). Breast cancer in elderly women: a different reality? Results from the NORA study. Ann Oncol.

[3] Yancik R, Wesley MN, Ries LA, Havlik RJ, Edwards BK, Yates JW (2001). Effect of age and comorbidity in postmenopausal breast cancer patients aged 55 years and older. JAMA.

[4] Hutchins LF, Unger JM, Crowley JJ, Coltman Jr CA, Albain KS (1999). Underrepresentation of patients 65 years of age or older in cancer-treatment trials. N Engl J Med.

[5] Lewis JH, Kilgore ML, Goldman DP (2003). Participation of patients 65 years of age or older in cancer clinical trials. J Clin Oncol.

[6] Quoix E, Zalcman G, Oster J-P (2011). Carboplatin and weekly paclitaxel doublet chemotherapy compared with monotherapy in elderly patients with advanced non-small-cell lung cancer: IFCT-0501 randomised, phase 3 trial. Lancet.

[7] Perou CM, Sørlie T, Eisen MB (2000). Molecular portraits of human breast tumours. Nature.

[8] Sørlie T, Perou CM, Tibshirani R (2001). Gene expression patterns of breast carcinomas distinguish tumor subclasses with clinical implications. Proc Natl Acad Sci U S A.

[9] Karnofsky DA, JH Burchenal (1949). The clinical evaluation of chemotherapeutic agents in cancer. Evaluation of Chemotherapeutic Agents.

[10] Oken MM, Creech RH, Tormey DC, Horton J, Davis TE, McFadden ET, Carbone PP (1982). Toxicity and response criteria of the Eastern Cooperative Oncology Group. Am J Clin Oncol.

[11] Sørensen J, Klee M, Palshof T, Hansen H (1993). Performance status assessment in cancer patients. An inter-observer variability study. Br J Cancer.

[12] Roila F, Lupattelli M, Sassi M (1991). Intra and interobserver variability in cancer patients' performance status assessed according to Karnofsky and ECOG scales. Ann Oncol.

[13] Lucci A, McCall LM, Beitsch PD (2007). Surgical complications associated with sentinel lymph node dissection (SLND) plus axillary lymph node dissection compared with SLND alone in the American College of Surgeons Oncology Group Trial Z0011. J Clin Oncol.

[14] Schijven M, Vingerhoets A, Rutten H, Nieuwenhuijzen G, Roumen R, Van Bussel M, Voogd A (2003). Comparison of morbidity between axillary lymph node dissection and sentinel node biopsy. Eur J Surg Oncol.

[15] Veronesi U, Paganelli G, Viale G (2003). A randomized comparison of sentinel-node biopsy with routine axillary dissection in breast cancer. N Engl J Med.

[16] Hladiuk M, Huchcroft S, Temple W, Schnurr BE (1992). Arm function after axillary dissection for breast cancer: a pilot study to provide parameter estimates. J Surg Oncol.

[17] Singletary SE, Shallenberger R, Guinee VF (1993). Breast cancer in the elderly. Ann Surg.

[18] Pettke E, Ilonzo N, Ayewah M, Tsantes S, Estabrook A, Ma AMT (2016). Short-term, postoperative breast cancer outcomes in patients with advanced age. Am J Surg.

[19] Oakley N, Dennison A, Shorthouse A (1996). A prospective audit of simple mastectomy under local anaesthesia. Eur J Surg Oncol.

[20] Hughes KS, Schnaper LA, Berry D (2004). Lumpectomy plus tamoxifen with or without irradiation in women 70 years of age or older with early breast cancer. N Engl J Med.

[21] EBCTC Group (2015). Aromatase inhibitors versus tamoxifen in early breast cancer: patient-level meta-analysis of the randomised trials. Lancet.

[22] Muss HB, Woolf S, Berry D (2005). Adjuvant chemotherapy in older and younger women with lymph node-positive breast cancer. JAMA.

[23] Owusu C, Lash TL, Silliman RA (2007). Effect of undertreatment on the disparity in age-related breast cancer-specific survival among older women. Breast Cancer Res Treat.

[24] Dragun A (2012). Adherence to treatment guidelines and survival in patients with early-stage breast cancer by age at diagnosis. Breast Dis Year Bk Q.

[25] Fentiman I, Christiaens M-R, Paridaens R (2003). Treatment of operable breast cancer in the elderly: a randomised clinical trial EORTC 10851 comparing tamoxifen alone with modified radical mastectomy. Eur J Cancer.

[26] Tang V, Zhao S, Boscardin J (2018). Functional status and survival after breast cancer surgery in nursing home residents. JAMA Surg.

[27] Woo J, Cheung A (1993). A survey of elderly people discharged from hospital. J Hong Kong Med Assoc.

[28] Senie RT, Rosen PP, Lesser ML, Snyder RE, Schottenfeld D, Duthie K (1980). Epidemiology of breast carcinoma II: factors related to the predominance of left‐sided disease. Cancer.

[29] Hayes SC, Janda M, Cornish B, Battistutta D, Newman B (2008). Lymphedema after breast cancer: incidence, risk factors, and effect on upper body function. J Clin Oncol.

[30] Tasmuth T, Von Smitten K, Kalso E (1996). Pain and other symptoms during the first year after radical and conservative surgery for breast cancer. Br J Cancer.

